# Placenta Percreta at 17 Weeks with Consecutive Hysterectomy: A Case Report and Review of the Literature

**DOI:** 10.1155/2012/734834

**Published:** 2012-10-02

**Authors:** Natasha Gupta, Anu Gupta, Marlene Green, Hyung Shik Kang, Josef Blankstein

**Affiliations:** Department of Obstetrics and Gynecology, Mount Sinai Hospital, Chicago, IL 60608, USA

## Abstract

Placenta percreta in early pregnancy is an extremely rare but life-threatening complication, for which very few cases have been reported in the literature worldwide, none from the United States. We report a patient with two previous cesarean deliveries, who presented with incomplete abortion at 17 weeks and underwent dilatation and curettage. She was found to have retained, adherent placenta that led to extensive hemorrhage, requiring emergency supracervical hysterectomy. Postoperative course was also complicated by severe consumption coagulopathy, necessitating reexploration after hysterectomy. Pathology revealed a placenta percreta. Patient lost more than 8000 cc blood through the 2 surgeries, received massive transfusions due to severe disseminated intravascular coagulopathy (DIC), and underwent a complicated surgery because of great difficulty in separating lower uterine segment and cervix from the bladder. Abnormal placentation in early pregnancy has increased in prevalence due to marked rise in cesarean deliveries and curettages in recent decades. We reviewed all reported cases of first and second trimester placenta percreta in the literature, to emphasize the early recognition of abnormal placentations in patients with risk factors, consider prenatal evaluation in such patients, anticipate complicated placental implantations during termination procedures, and prevent associated maternal morbidity and mortality.

## 1. Introduction

Abnormal placentation during pregnancy is categorized into accreta, increta, and percreta in the order of increasing severity and increasing invasion of placental villi through the uterine wall. Accreta involves penetration of placental tissue beyond the endometrial lining, into the myometrium, increta is characterized by deep myometrial invasion, and percreta involves placental penetration through the uterine serosa and into adjacent organs in some cases. Placenta percreta is the most severe form of placentation defect, noted very infrequently in first or second trimester. It can be a lethal problem when encountered during a dilatation and evacuation or curettage performed for the termination of first or second trimester pregnancy loss, leading to extensive hemorrhage within minutes of initiating the procedure. Individuals with risk factors for placenta accreta or percreta at term should be investigated early in pregnancy. Case in point exemplifies the need of aggressive evaluation for placentation defects in patients with history of previous cesarean deliveries or curettages and encourages the physician to employ more conservative methods like uterine artery embolization or methotrexate prior to curettage for termination.

## 2. Case Presentation

We report a 21-year-old Hispanic female gravida three para two, at 17 weeks gestational age who presented to emergency room with abdominal pain and vaginal bleeding. She complained of having spontaneously expelled the fetus at home. Upon examination, her cervical os was noted to be open with active, moderate vaginal bleeding and retained placenta inside. She had a history of prior two cesarean sections and no medical problems. Her vital signs on admission were stable and hemoglobin was 11.6 mg/dL.

She was recommended a dilatation and curettage (D and C) procedure due to retained placenta. The patient was noted to have persistent, active bleeding which was unresponsive to uterine bimanual massage, oxytocin infusion, hemabate, and methergine. At that time, patient was also hypotensive and a decision was made to perform an exploratory laparotomy with possible hysterectomy. During laparotomy, placenta was found to be densely adherent to the lower uterine segment and posterior wall of the bladder. We amputated the uterus just above the lower uterine segment, due to great difficulty in separating bladder from the lower uterine segment and packed the cervix and lower uterine segment with Surgicel. The patient was noted to have diffuse oozing from the pedicles and disseminated intravascular coagulopathy (DIC) was suspected. Methylene blue dye was used to ensure that bladder was intact. At this time, we terminated the procedure and the laparotomy site was closed. The patient received 6 units of packed blood and 2 units of fresh frozen plasma during this surgery, with a blood loss of 3000 cc. Post-operative coagulation profile was PT 18.95, APTT 40.3, Fibrinogen 135, Hemoglobin 10.9, and platelets of 61.

The patient was then carefully monitored in surgical intensive care unit and was also taken for a uterine artery embolization due to persistent bleeding. Overnight the patient received 6 units of packed blood, 4 units of frozen plasma, crystalloids, and albumin. 

Next morning, she still had moderate vaginal bleeding with labile vital signs. Her DIC was noted to be more under control. We decided to perform a reexploration at that time. During this second surgery, residual lower uterine segment and cervix were noted to be enlarged, with active bleeding. We sharply separated the bladder from the lower uterine segment, followed by careful resection of the lower uterine segment and the cervix. Excellent hemostasis was ensured. Bladder integrity was confirmed with indigo carmine dye test resulting in no dye extravasation. The estimated blood loss was 5700 cc during this second surgery. Her postop course remained uneventful. She was discharged home on postoperative day 6 without any complications. 

Histopathology showed placental villi extending deeply into and through the myometrium of the lower uterine segment, suggestive of placenta percreta in the lower uterine segment.

## 3. Discussion

Placenta percreta is defined as an abnormal adherence of placenta to the uterine wall secondary to total or partial absence of the decidua basalis. Histologically, it is characterized by chorionic villi penetrating through the myometrium into the uterine serosa and may infiltrate the surrounding organs such as urinary bladder and bowel. Separation of such a placenta from the myometrium can result in fatal hemorrhage as trophoblastic tissue is very vascular. The incidence of accreta has increased from 1 in 30,000 to 1 in 2500 in the past 50 years, which is attributable to increase in the rate of cesarean deliveries [1].

 The risk factors for defective placentation include previous uterine surgeries like cesarean section [2], myomectomy and curettages and also endometrium involvements with endometritis or pyometra, abnormal placental localization, and submucosal fibroids. In majority of the studies, predisposing factors for abnormal placentation in early pregnancy were related to thinning of the endometrium at the site of uterine scars from previous cesarean sections. Clark and colleagues studied the relationship between previous cesarean section and placental abnormalities and noted that risk of placental disorders including placenta previa increases with the number of previous cesarean sections [3]. Esmans et al. from Belgium reported a case of rupture of an unscarred uterus from placenta percreta in a woman with a history of manual extraction of placenta in previous pregnancy [4].

In our detailed analysis of the literature, we found very few cases of placenta percreta in first or second trimester of pregnancy, worldwide and none in the United States. However, placenta accreta has been reported more commonly in early pregnancy as compared to percreta. Rashbaum et al. noted a prevalence of 0.04% of clinical placenta accreta encountered during dilatation and evacuations (D and E) in second trimester, same as that of clinical placenta accreta diagnosed in third trimester. They reviewed 7 cases of placenta accreta encountered during D and E in second trimester, all of which required hysterectomy [5]. We found 4 cases of early placenta percreta reported from Japan, Germany, Greece, and France [6–9]. Komiya et al. from Japan described a case of incomplete abortion at 21 weeks, taken for curettage which resulted in profuse vaginal bleeding and hemorrhagic shock [6]. The patient received vasopressin, dobutamine, norepinephrine to maintain cardiocirculatory function, also a large amount of i.v fluids, packed cells, and finally a hysterectomy. Höpker et al. from Germany reported a similar presentation at 10 weeks gestation in a patient who was suspected to have molar pregnancy on sonogram and thus received CT, MRI as part of the work-up, all of these showed trophoblastic infiltration through the myometrium into the serosa [8]. A D and C was undertaken for suspected molar, which resulted in severe hemorrhage, and the patient needed hysterectomy despite uterine artery ligation. Pathology revealed placenta percreta, without any evidence of hydatiform mole. Papadakis and Christodoulou from Greece described another early placenta percreta that went unrecognized and required hysterectomy after a curettage resulted in heavy bleeding [9]. Also, Pont et al. from France noted a case of acute abdomen and hemoperitoneum at 13 weeks gestation [7]. Patient underwent laparotomy which diagnosed the placenta percreta and a hysterectomy was performed. All these cases describe unrecognized first or second trimester placenta percreta that led to extensive blood loss, lengthy operations, and caused considerable maternal morbidity.

Spontaneous rupture of uterus in early pregnancy is another lethal complication of early placenta percreta leading to significant hemoperitoneum and shock, thus necessitating hysterectomy. A few such cases of spontaneous rupture of uterus due to placenta percreta have been reported in countries other than USA, like Japan, Turkey, Mexico, and Germany [10–14]. All these cases noted either previous cesarean deliveries or curettages in their patients, with the exception of Kinoshita et al. from Japan, who reported a case of spontaneous rupture of uterus due to placenta percreta in the patient's first pregnancy without a background of any risk factors [12]. All these patients underwent hysterectomy. This catastrophic complication from early percreta occurs due to thinning of the myometrium caused by invasion of the placental villi into the myometrium, at the site of placental implantation (particularly at previous scar site) leading to rupture of the uterus. 

The modern physician needs to be aware of both these types of presentations of early placenta percreta—(1) where percreta goes unrecognized and is diagnosed only during a curettage procedure, when heavy vaginal bleeding occurs. (2) where a patient presents in first or second trimester with acute abdomen and hemorrhagic shock, resulting from spontaneous rupture of the uterus. 

In this era of increasing cesarean deliveries, emphasis should be made on correct prenatal diagnosis of implantation defect, to prevent maternal morbidity in face of an incomplete abortion. Signs suggestive of placenta accreta/percreta may be present as early as the first trimester. Some authors have suggested a relationship between abnormal placentation and unexplained, elevated MSAFP in maternal blood. Some of the imaging tools recommended in the evaluation of placental invasion include—Doppler Sonography, gray-scale sonography, and magnetic resonance imaging (MRI) [15]. They identify thinned decidual endometrium, thinned myometrium, and placental extension into the myometrium. Sonograms can detect loss of hypoechoicity of the myometrium between the bladder and the placental wall. Also, visualization of intraplacental sonolucent spaces also referred to as venous lakes, adjacent to the involved uterine-wall is strongly suggestive of placenta accreta. The diagnostic sensitivity and specificity approaches 85 to 90 percent with experienced sonographers. MRI can be used if sonogram findings are uncertain. Signs such as uterine bulging into the bladder, heterogeneous signal intensity within the placenta, and the presence of intra placental bands may predict accreta. These imaging modalities have been used with mixed success, for evaluation of patients with placenta previa to diagnose placental accreta. Cystoscopy can be used to detect bladder involvement where placenta percreta is strongly suspected. 

In our survey of the literature, all of the cases presenting with defective placentation were treated with hysterectomy. Tocce et al. recommended performing a scheduled hysterectomy for second trimester abortion in a patient with placenta accrete [16]. Such a plan of care is possible when a diagnosis of placenta accreta or percreta is established early in the pregnancy with the use of color Doppler ultrasonography, MRI, and cystoscopy, in a setting of risk factors. Traditionally, hysterectomy has been the choice of modality, but it may be avoided by an early diagnosis of percreta. Yu et al. reviewed 31 cases of second trimester placenta accreta and employed conservative methods like uterine artery embolization and hysteroscopy with lesion resection, mostly in combination [17]. None of their patients needed hysterectomy. Some authors have suggested the use of methotrexate, in combination with curettage or embolization. These methods have been recommended by various authors based on their management of placenta accreta in second trimester or at full term. Same principles of diagnosis and treatment may also be employed for management of placenta percreta. Conservative treatment options although help to preserve the uterus, are not without associated risks. Help from urologists in a timely manner is recommended to avoid urinary complications. However, there is no available literature to guide the adequate diagnostic and treatment options of early placenta percreta, due to the rarity of this condition.

## 4. Conclusion

Placenta percreta is a greatly feared obstetric complication, even when presenting at full-term delivery as it may lead to emergency hysterectomy, consumptive coagulopathy, extensive hemorrhage, and urinary complications. There is an abnormal connection between trophoblasts and uterine myometrium because of improper development of the decidua. Manual separation of placenta in such a case causes life threatening intraoperative bleeding and subsequent development of DIC, acute respiratory distress syndrome, renal failure, and even death. It is seldom seen in early pregnancy. Review of the literature has shown only a few cases of percreta at an early gestation. High index of suspicion is necessary in patients with risk factors. As we step into an era where cesarean section rate is on its hike, modern obstetrician needs to anticipate the likelihood of an early percreta encountered during a routine curettage. Hysterectomy seems to be the only adequate treatment available thus far particularly due to bladder involvement, but uterus sparing options need to be explored further.
